# The Predictors of Osteoarthritis Among U.S. Adults: National Health and Nutrition Examination Survey, 2005 to 2018

**DOI:** 10.7759/cureus.63469

**Published:** 2024-06-29

**Authors:** Ayobami S Ogunsola, Arman C Hlas, Michael C Marinier, Jacob Elkins

**Affiliations:** 1 Department of Orthopaedics and Rehabilitation, University of Iowa Hospitals and Clinics, Iowa City, USA

**Keywords:** obesity, united states, adults, health predictors, nhanes, osteoarthritis

## Abstract

Background

Osteoarthritis (OA) is a leading cause of disability in the United States (U.S.) population, and its prevalence continues to rise. Traditionally, extreme joint loading was described as the leading cause of OA; however, recent studies suggest OA may arise from more complex mechanisms. This study aimed to identify the association between OA and various health predictors among U.S. adults.

Methods

National Health and Nutrition Examination Survey (NHANES) data of adult participants from 2005 to 2018 was reviewed. OA diagnosis was patient-reported, and other health variables were assessed based on patient-reported, laboratory, and examination data. A multivariable survey logistic regression model was used to estimate adjusted odds ratios and confidence intervals (95% CIs). Stratified analysis based on BMI category was additionally performed to assess the modifying effect of obesity on the association between OA and health predictors.

Results

A total of 42,143 participants were included in this study. OA prevalence was highest in patients ages $\geq$ 65 years, females, non-obese individuals, non-Hispanic Whites, and those with at least college education. After controlling for multiple confounding demographic variables and comorbidities, the odds of OA increased with aging, female sex, obesity, high cholesterol, hypertension, diabetes, depression, and thyroid disease. Non-Hispanic White patients and those with less than a high school education also had higher odds of OA. After stratified analysis, aging, female sex, and severe depression demonstrated similar associations with OA across each BMI strata. Having at least a college-level education additionally conferred a similar association with OA across each BMI strata.

Conclusion

The odds of OA increased with aging, female sex, obesity, less than high school education, high cholesterol, hypertension, diabetes, depression, and thyroid disease. Further studies are needed to characterize the mechanisms of these associations. Given the myriad of factors that influence OA development and progression, the utilization of multidisciplinary and holistic care of OA patients is recommended to limit the influence of other health predictors and reduce ensuing pain, disability, and other complications that result from OA.

## Introduction

Osteoarthritis (OA) is the most common joint disorder and leading cause of disability in the United States (U.S.) population [1,2]. The chronicity of OA leads to significant pain and reduced quality of life in affected patients, impacting overall health which can lead to the development or worsening of other comorbid medical conditions [3-5]. Current estimates project that OA accounts for $80 billion in total U.S. healthcare spending annually, leading to increased financial burden on both patients and the health system broadly [6]. The impact of OA remains a concern as it grows in prevalence, with a reported 113% increase in global OA prevalence from 1990 to 2019 [7].

Historically, OA has been thought to arise from excessive joint loading and degeneration of articular surfaces over time. However, recent studies suggest the mechanism of OA may be more complex due to the occurrence of OA on non-weightbearing joints, with strong risk factors including age, sex, obesity, and previous joint injury [4]. Obesity, quantified through body mass index (BMI), has been theorized to impact OA by increasing joint load and inflammatory cytokine signaling, limiting mobility, and exacerbating other related health conditions that impact OA [8,9]. Other patient factors such as race/ethnicity, cigarette use, physical activity level, comorbid medical conditions such as hypertension and diabetes, and indicators of socioeconomic status (SES) including education and income levels have also been implicated with OA progression [10-15].

While obesity remains a well-characterized risk factor for OA development and progression, less is currently known about other patient factors and how they relate to OA in context of their obesity status. This study primarily aims to identify the association between OA and various health predictors among U.S. adults.

## Materials and methods

Data collection

This study extracted data from the National Health and Nutrition Examination Survey (NHANES) from 2005 to 2018 for its analysis. NHANES is a cross-sectional survey based on a nationally representative sample of the U.S. population across all age groups. Individual demographic, behavioral, examination, and health data are collected from participants based on personal interviews, laboratory data, and standardized physical examinations [16]. NHANES data is publicly available and de-identified prior to release, therefore this study was exempt from Institutional Review Board review. 

Variable definitions

The self-reported age variable in this study was restricted to 18 years and older. Participants were grouped into age categories of $&lt;$ 45, 45-64, and $\geq$ 65 years of age. Patients were classified with OA according to two patient-reported variables: “Has a doctor or other health professional ever told you that you have arthritis?” and “Which type of arthritis was it?”. BMI was grouped into categories of $&lt;$ 30 kg/m^2^, 30-40 kg/m^2^, and $\geq$ 40 kg/m^2^. Total cholesterol was grouped as “normal” or “high” based on levels of $&lt;$ 240 mg/dL or $\geq$ 240 mg/dL, respectively. Participants were classified as diabetic if they had either a physician diagnosis of diabetes or a fasting blood glucose of $\geq$ 200mg/dL. Patient-reported physician diagnosis of hypertension, systolic blood pressure, and diastolic blood pressure measurements were used to classify the hypertension variable. Depression was classified in study participants as “None/Minimal”, “Mild/Moderate”, and “Severe” using Patient Health Questionnaire-9 (PHQ-9) scores of $&lt;$ 5, 5-14, and $\geq$ 15, respectively. Thyroid disease was classified as patient-reported physician diagnosis. Other variables included in this study were patient-reported sex (male or female), race/ethnicity (non-Hispanic White, non-Hispanic Black, Hispanic, or other), education level (less than high school, high school, or at least college), and cigarette use (yes or no). 

Statistical analysis

Descriptive statistics were completed to analyze the distribution of study variables. Weighted percentages were estimated for categorical variables and measures of central tendency (mean and standard deviation) were used to estimate the distribution of continuous variables. The crude odds ratio between categorical variables was assessed with a chi-square statistic and a 5% margin of error. 

To assess the association between OA and other health variables, we performed the stepwise model selection process to identify the most significant predictors of OA while minimizing model complexity. We fitted a multivariate survey logistic regression model with OA as the outcome variable and adjusted for age, sex, BMI, race/ethnicity, cigarette use, education level, cholesterol level, hypertension, diabetes, depression, and thyroid disease. To evaluate the potential modifying effect of obesity on the association between OA and health predictors, we performed stratified analysis based on BMI category. This analysis offered a more nuanced understanding of the associations between health predictors and OA within different BMI categories. Overall, this model accounted for the complex survey design: weighting, clustering, and sample stratification. All statistical analyses were conducted using SAS (SAS Institute; Cary, NC, USA) [17].

## Results

**Figure 1:**
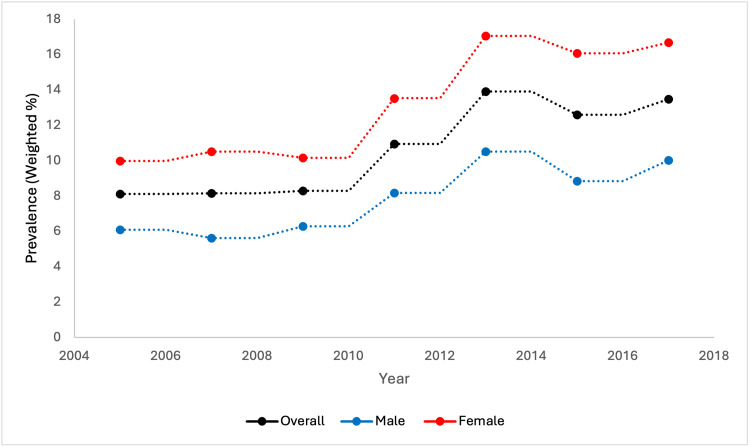
Biannual prevalence of osteoarthritis in U.S. adults from the National Health and Nutrition Examination Survey (NHANES).

The total number of study participants in our analysis was 42,143. The majority were less than 45 years of age (n = 19,221; 47.81%), female (n = 21,685; 51.76%), BMI $&lt;$ 30 kg/m^2^ (n = 27,414; 65.25%), non-Hispanic White (n = 17,194; 66.31%), with at least a college-level education (n = 20,496; 58.31%). 4,021 patients were identified with osteoarthritis (10.87%). Few participants reported cigarette use (n = 8,215; 19.84%), high cholesterol (n = 15,277; 36.40%), hypertension (n = 13,632; 29.92%), diabetes (n = 7,503; 14.98%), or thyroid disease (n = 4,010; 10.44%). Most respondents had minimal to no symptoms of depression (n = 33,013; 79.49%) (Table 1). Figure 1 illustrates the biannual trend of OA prevalence with additional stratification by sex. 

**Table 1:** Descriptive characteristics of variables in U.S. adults from the National Health and Nutrition Examination Survey (NHANES) (2005–2018). BMI: Body Mass Index.

Characteristic	Patient total (N, %)	Weighted %
Age (years)		
$\small &lt; 45$	19221 (45.61)	47.81
$\small 45 - 64$	13220 (31.37)	34.36
$\small \geq 65$	9702 (23.02)	17.83
Sex		
Male	20458 (48.54)	48.24
Female	21685 (51.46)	51.76
BMI (kg/m^2^)		
$\small &lt; 30$	27414 (65.05)	65.25
$\small 30 - 40$	11917 (28.28)	28.19
$\small > 40$	2812 (6.67)	6.56
Race/ethnicity		
Non-Hispanic White	17194 (40.80)	66.31
Non-Hispanic Black	9253 (21.96)	11.52
Hispanic	10843 (25.73)	14.32
Other	2455 (5.83)	7.85
Cigarette use		
Yes	8215 (19.49)	19.84
No	9527 (22.61)	23.60
Education level		
Less than High School	10090 (23.94)	15.77
High School	9102 (21.60)	22.51
At least College	20496 (48.63)	58.31
Total cholesterol (mg/dl)		
$\small \geq 240$	15277 (36.25)	36.40
$\small &lt; 240$	26866 (63.75)	63.60
Hypertension		
Yes	13632 (32.35)	29.92
No	23427 (55.59)	57.78
Diabetes mellitus		
Yes	7503 (17.80)	14.98
No	34640 (82.20)	85.02
Osteoarthritis		
Yes	4021 (9.54)	10.87
No	28947 (68.96)	71.95
Depression		
None/minimal	33013 (78.34)	79.49
Mild/moderate	7837 (18.60)	17.92
Severe	1293 (3.07)	2.59
Thyroid disease		
Yes	4010 (9.52)	10.44
No	35644 (84.58)	86.08

Bivariate analysis of OA and selected predictors

A majority of OA patients were 65 years of age or older (n = 2,111; 45.23%), female (n = 2,571; 64.49%), BMI $&lt;$ 30 kg/m^2^ (n = 2,195; 54.71%), non-Hispanic White (n = 2,475; 82.47%), with at least a college-level education (n = 2,271; 24.28%). Most respondents with OA reported a history of hypertension (n = 2,285; 53.22%) and diabetes (n = 2,285; 53.22%). Prior to adjusting for other variables, the odds of OA increased with aging, female sex, obesity, high cholesterol, hypertension, diabetes, depression, and thyroid disease (Table 2). 

**Table 2:** Bivariate analysis of osteoarthritis and selected predictors in U.S. adults from the National Health and Nutrition Examination Survey (NHANES) (2005–2018). BMI: Body Mass Index, OA: osteoarthritis, OR: odds ratio

Variables	Patients with OA (Weighted %)	Patients without OA (Weighted %)	Crude OR (95% CI)
Age (years)			
$\small &lt; 45$	396 (11.17)	15503 (56.75)	Reference
$\small 45 - 64$	1514 (43.60)	8861 (32.11)	2.69 (2.49 – 2.91)
$\small > 65$	2111 (45.23)	4583 (11.15)	5.62 (5.23 – 6.04)
Sex			
Male	1450 (35.51)	14844 (50.96)	Reference
Female	2571 (64.49)	14103 (49.04)	1.42 (1.35 – 1.49)
BMI (kg/m^2^)			
$\small &lt; 30$	2195 (54.71)	19639 (50.10)	Reference
$\small 30 - 40$	1383 (34.80)	7752 (26.41)	1.43 (1.35 – 1.52)
$\small > 40$	443 (10.49)	1556 (4.11)	2.03 (1.86 – 2.23)
Race/ethnicity			
Non-Hispanic White	2475 (82.47)	10986 (63.50)	Reference
Non-Hispanic Black	649 (6.68)	6193 (11.75)	0.87 (0.81 – 0.93)
Hispanics	596 (5.46)	7928 (16.10)	0.56 (0.52 – 0.61)
Other	301 (5.39)	3840 (8.65)	0.60 (0.54 – 0.67)
Cigarette use			
No	669 (16.04)	5978 (20.48)	Reference
Yes	1417 (35.67)	5900 (21.27)	0.69 (0.63 – 0.75)
Education level			
Less than High School	830 (12.94)	6970 (11.91)	Reference
High School	914 (22.74)	6474 (22.64)	0.89 (0.82 – 0.96)
At least College	2271 (24.28)	15467 (61.77)	0.70 (0.65 – 0.76)
Total cholesterol (mg/dl)			
$\small &lt; 240$	2308 (58.03)	9277 (31.64)	Reference
$\small \geq 240$	1713 (41.97)	19670 (68.37)	1.88 (1.77 – 1.99)
Hypertension			
No	1283 (34.95)	17603 (63.41)	Reference
Yes	2285 (53.22)	7604 (23.72)	2.55 (2.40 – 2.72)
Diabetes mellitus			
No	1283 (34.95)	24924 (88.54)	Reference
Yes	2285 (53.22)	4023 (11.46)	2.25 (2.08 – 2.43)
Depression			
None/minimal	2807 (71.79)	23682 (82.58)	Reference
Mild/moderate	1001 (24.30)	4639 (15.52)	1.75 (1.64 – 1.87)
Severe	203 (3.91)	626 (1.90)	2.57 (2.31 – 2.87)
Thyroid Disease			
No	3115 (76.14)	26836 (92.28)	Reference
Yes	895 (23.71)	2065 (7.60)	2.69 (2.50 – 2.90)

Association between OA and selected predictors

After controlling for confounding demographic variables and comorbidities, the odds of OA increased with aging (45 - 64 years: Adjusted Odds Ratios [aOR]= 3.79, confidence interval [95% CI]: 3.34 - 4.29; $\geq$ 65 years: aOR= 7.11, [95% CI]: 6.18 - 8.18), obesity (30 - 40 kg/m^2^: aOR= 1.31, [95% CI]: 1.19 - 1.45; $>$ 40 kg/m^2^: aOR= 1.77, [95% CI]: 1.52 - 2.06), high cholesterol (aOR= 1.18, [95% CI]: 1.08 - 1.30), hypertension (aOR= 1.40, [95% CI]: 1.28 - 1.54), diabetes (aOR= 1.31, [95% CI]: 1.14 - 1.50), depression (Mild/Moderate: aOR = 1.72, [95% CI]: 1.55 - 1.92; Severe: aOR= 2.41, [95% CI]: 2.03 - 2.85), and thyroid disease (aOR= 1.24, [95% CI]: 1.10 - 1.40). Female (aOR= 1.44, [95% CI]: 1.31 - 1.58) respondents had higher odds of OA compared to male respondents, and participants with a high school education (aOR= 0.83, [95% CI]: 0.74 - 0.93) or at least college education (aOR= 0.73, [95% CI]: 0.65 - 0.81) had lower odds of OA compared to those with less than a high school education. Non-Hispanic Black (aOR= 0.87, [95% CI]: 0.78 - 0.98) and Hispanic (aOR= 0.61, [95% CI]: 0.54 - 0.69) respondents additionally had lower odds of OA compared to non-Hispanic Whites. Cigarette use (aOR= 1.09, [95% CI]: 0.99 - 1.20) demonstrated no significant association with OA in the overall model (Table 3). 

**Table 3:** Association between osteoarthritis and selected predictors in U.S. adults from the National Health and Nutrition Examination Survey (NHANES) (2005-2018). BMI: Body Mass Index, aOR: adjusted odds ratio

Variable	Overall aOR (95% CI)	BMI < 30 aOR (95% CI)	BMI 30 – 40 aOR (95% CI)	BMI > 40 aOR (95% CI)
Age (years)				
$\small &lt; 45$	Reference	Reference	Reference	Reference
$\small 45 - 64$	3.79 (3.34 – 4.29)	4.05 (3.45 – 4.75)	3.38 (2.67 – 4.27)	4.48 (3.05 – 6.59)
$\small \geq 65$	7.11 (6.18 – 8.18)	7.47 (6.27 – 8.88)	6.55 (4.99 – 8.58)	7.14 (4.20 – 12.45)
Sex				
Male	Reference	Reference	Reference	Reference
Female	1.44 (1.31 – 1.58)	1.42 (1.25 – 1.60)	1.34 (1.16 – 1.55)	2.29 (1.67 – 3.13)
BMI (kg/m^2^)				
$\small &lt; 30$	Reference	-	-	-
$\small 30 - 40$	1.31 (1.19 – 1.45)	-	-	-
$\small > 40$	1.77 (1.52 – 2.06)	-	-	-
Race/ethnicity				
Non-Hispanic White	Reference	Reference	Reference	Reference
Non-Hispanic Black	0.87 (0.78 – 0.98)	0.79 (0.69 – 0.91)	0.99 (0.84 – 1.17)	0.87 (0.65 – 1.15)
Hispanic	0.61 (0.54 – 0.69)	0.57 (0.49 – 0.67)	0.62 (0.52 – 0.75)	0.81 (0.52 – 1.25)
Other	0.82 (0.66 – 1.03)	0.69 (0.51 – 0.93)	0.69 (0.57 – 0.83)	0.61 (0.26 – 1.42)
Cigarette use				
No	Reference	Reference	Reference	Reference
Yes	1.09 (0.99 – 1.20)	1.17 (1.03 – 1.33)	0.94 (0.80 – 1.11)	1.10 (0.78 – 1.56)
Education level				
Less than High School	Reference	Reference	Reference	Reference
High School	0.83 (0.74 – 0.93)	0.88 (0.75 – 1.02)	0.81 (0.66 – 0.99)	0.60 (0.39 – 0.93)
At least College	0.73 (0.65 – 0.81)	0.81 (0.71 – 0.94)	0.69 (0.57 – 0.83)	0.35 (0.23 – 0.55)
Total cholesterol (mg/dl)				
$\small &lt; 240$	Reference	Reference	Reference	Reference
$\small \geq 240$	1.18 (1.08 – 1.30)	1.09 (0.97 – 1.23)	1.35 (1.16 – 1.57)	1.39 (1.01 – 1.93)
Hypertension				
No	Reference	Reference	Reference	Reference
Yes	1.40 (1.28 – 1.54)	1.53 (1.34 – 1.75)	1.17 (0.97 – 1.41)	1.56 (1.10 – 2.20)
Diabetes mellitus				
No	Reference	Reference	Reference	Reference
Yes	1.31 (1.14 – 1.50)	1.45 (1.21 – 1.74)	1.18 (0.96 – 1.45)	1.33 (0.96 – 1.84)
Depression				
None/minimal	Reference	Reference	Reference	Reference
Mild/moderate	1.72 (1.55 – 1.92)	1.94 (1.70 – 2.21)	1.59 (1.32 – 1.92)	1.19 (0.81 – 1.73)
Severe	2.41 (2.03 – 2.85)	2.44 (1.88 – 3.17)	2.35 (1.61 – 3.41)	2.25 (1.39 – 3.66)
Thyroid disease				
No	Reference	Reference	Reference	Reference
Yes	1.24 (1.10 – 1.40)	1.45 (1.27 – 1.66)	1.14 (0.88 – 1.47)	0.72 (0.49 – 1.08)

After stratification according to BMI category, aging, female sex, and severe depression demonstrated similar associations with OA across each BMI strata. At least college education conferred a similar association with OA across each BMI strata, however high school education demonstrated a similar association only in BMI categories 30-40 kg/m^2^ and $>$ 40 kg/m^2^. Total cholesterol $\geq$ 240 mg/dL demonstrated direct association with OA in those with BMI category of 30-40 kg/m^2^ or $>$ 40 kg/m^2^, and hypertension demonstrated similar association in participants belonging to BMI category $&lt;$ 30 kg/m^2^ or $>$ 40 kg/m^2^. Diabetes, thyroid disease, and cigarette use showed direct association with OA in those with BMI $&lt;$ 30 kg/m^2^. The protective effect observed among non-Hispanic Blacks in BMI category 30-40 kg/m^2^ and among all racial categories in BMI category $>$ 40 kg/m2 was lost (Table 3).

## Discussion

Using data from a nationally representative sample of U.S. residents from 2005 to 2018, OA prevalence was found to vary based on multiple modifiable and non-modifiable health predictors. Overall, OA prevalence was highest in patients ages 65 years or older, females, non-obese individuals, non-Hispanic Whites, and those with at least college education. After adjusting for potential confounders in the overall model, aging, obesity, female sex, high cholesterol, hypertension, diabetes, depression, and thyroid disease were associated with increased odds of OA among study participants. Non-Hispanic Black and Hispanic respondents had reduced odds of OA compared to non-Hispanic White respondents. Participants with a high school and college education also had reduced odds of OA compared to those with less than a high school education. After stratification based on BMI, the direct association between OA and increasing age category, female sex, and severe depression persisted across all BMI strata. At least college education conferred a similar association with OA across each BMI strata as well. However, high cholesterol demonstrated association with OA only in those with BMI 30-40 kg/m^2^ or $>$ 40 kg/m^2^, and hypertension demonstrated association in those with BMI $&lt;$ 30 kg/m2 or $>$ 40 kg/m^2^. Diabetes, thyroid disease, and cigarette use showed association with OA only in respondents with BMI $&lt;$ 30 kg/m^2^. The observed effect with race in the overall model was lost among non-Hispanic Blacks in BMI category 30-40 kg/m^2^ and among all racial categories in BMI category $>$ 40 kg/m^2^.

As for the non-modifiable health predictors included in this study, our findings corroborate the well-known relationship between increasing age and female sex with OA development [4]. OA was more prevalent among older and female participants in our study; after adjusting for numerous health predictors, increasing age and female sex were also found to increase odds of OA across all BMI strata. These relationships have been previously elucidated: musculoskeletal aging increases susceptibility to OA through alterations in chondrocyte metabolism and loss of normal bone and articular surface integrity, in addition to other biochemical aging processes affecting joint structure and function [18]. Furthermore, females have been thought to have increased rates of OA due to gender-related differences in skeletal morphometry, kinematics, levels of circulating sex hormones, and utilization of health care resources broadly [19]. Racial and ethnic classifications have also been associated with variable OA prevalence. This study identified non-Hispanic Whites as the self-identified group with the highest prevalence of OA, and non-Hispanic Blacks and Hispanics were found to have reduced odds of OA compared to non-Hispanic White respondents. In contrast to these findings, several U.S.-based studies have indicated African Americans have increased prevalence of specific radiographic features of hip OA and increased prevalence of radiographic and symptomatic knee OA compared to white populations [20-23]. Differences in OA severity and phenotype, genetic makeup, and pain processing have also been shown to impact OA rates among different racial/ethnic groups [10].

Multiple modifiable health predictors including obesity, high cholesterol, hypertension, diabetes, depression, and thyroid disease were associated with increased odds of OA. Like any disease state, a mechanistic relationship likely exists. Depression may increase risk of OA through changes in appetite, hormone dysregulation, or the use of antidepressant medications [24]. Subchondral ischemia has been identified as one of the leading mechanisms of OA in hypertensive patients, and increased lipid deposition in chondrocytes has also been thought to contribute to OA development [25,26]. Metabolic OA, an emerging subtype of OA, combines the effects of truncal obesity, hypertension, hyperlipidemia, and diabetes [27]. Genetic defects affecting local thyroid hormone availability in joint tissues and autoimmune thyroid disease has additionally been implicated with development of OA [28,29].

In contrast to other disease states, the relationship between tobacco use and OA is more obscure with previous research producing conflicting results [11,30,31]. This study found that cigarette use increased the odds of OA in those with BMI $&lt;$ 30 kg/m^2^, however did not display a significant association with OA in the overall model or at other BMI levels. Amin et al discovered that men with knee OA who smoked experienced greater cartilage loss and more severe pain than men who did not smoke [32]. Authors proposed that smoking may contribute to OA by causing chondrocyte dysfunction and altered cell metabolism, or by increasing carbon monoxide levels in the blood leading to impaired blood oxygenation and ineffective cartilage repair. Similarly, smoking has been shown to accelerate cartilage loss in those with family history of OA [33]. However, other studies propose smoking may have a protective effect against OA development. A 1988 study using data from the first U.S. Health and Nutrition Examination Survey unexpectedly found a protective association between smoking and OA, despite adjusting for patient age, sex, and weight [34]. This was later supported with data from the Framingham Osteoarthritis study: smokers had lower rates of OA than non-smokers, and nicotine was thought to possibly have a protective effect on articular cartilage [35]. More recent studies have also suggested an inverse association between smoking and OA [36]. Other research has demonstrated smoking has no protective effect against OA [37,38]. Similar to the current body of literature, our findings suggest that the relationship between smoking and OA is obscure and controversial. Further research is needed to clarify the relationship between smoking, OA, and other patient-related factors.

Social determinants of health have a well-known impact on many disease states, with links to modifiable factors like education and socioeconomic status as well as non-modifiable factors such as race and ethnicity. This study revealed that having at least high school education may have a protective effect against OA compared to those with less than high school education. This finding suggesting increased odds of OA among those with less than high school education may be attributed to field of work, where traditionally workers with lower education levels filled service and labor roles with greater physical demands [39]. Utilization of healthcare resources and socioeconomic status have also been shown to play a role in OA development among people of different educational levels [10,15].

Obesity is a strong risk factor for OA due to combined effects of excess mechanical loading and induction of a proinflammatory state resulting in chronic inflammation and joint degradation over time [27]. After stratifying respondents according to individual BMI, only increasing age, female sex, and severe depression were associated with greater odds of OA across each BMI strata. At least college education additionally conferred a similar association with OA across each BMI strata. This may be in part due to a strongly associated relationship between these specific health predictors and OA. The absence of other health predictors demonstrating similar associations across each category of BMI may be due to a modifying effect of obesity on the association between included health predictors and OA; however, significance in the overall model as well as across varying categories of BMI suggests an association likely still exists between these variables. These findings reinforce current frameworks of a complex and multifactorial pathogenesis of OA. Further studies are needed to characterize this complicated mechanism. This study’s findings support the utilization of interdisciplinary and holistic care of OA patients to limit the influence of medical comorbidities and other health predictors and reduce the ensuing pain, disability, and other complications resulting from OA. 

This study is not without limitations. Given the cross-sectional restraints of this study, we could only demonstrate association and not determine causation or directionality in the relationship between OA and included health predictors. Some NHANES data is additionally self-reported and thus susceptible to information bias, which could lead to potential misclassification. Lastly, the diagnosis of OA was generalized; therefore, it is unclear which location within the body (i.e. weightbearing versus non-weightbearing) was affected by disease in study subjects. 

## Conclusions

This study extracted data from a large and nationally representative sample of the U.S. adult population to examine the association between OA and various health predictors. After adjusting for multiple confounding variables and comorbidities, aging, female sex, obesity, less than high school education, high cholesterol, hypertension, diabetes, depression, and thyroid disease were associated with increased odds of OA. Given the myriad of factors that influence OA development and progression, the utilization of multidisciplinary and holistic care of OA patients is recommended to limit the influence of other health predictors and reduce the ensuing pain, disability, and other complications that result from OA. 
